# A network model for angiogenesis in ovarian cancer

**DOI:** 10.1186/s12859-015-0551-y

**Published:** 2015-04-11

**Authors:** Kimberly Glass, John Quackenbush, Dimitrios Spentzos, Benjamin Haibe-Kains, Guo-Cheng Yuan

**Affiliations:** 1 Dana-Farber Cancer Institute, Boston, MA USA; 2 Harvard School of Public Health, Boston, MA USA; 3 Brigham and Women’s Hospital, Boston, MA USA; 4 Beth Israel Deaconess Medical Center, Harvard Medical School, Boston, MA USA; 5 Princess Margaret Cancer Center, University Health Network, Toronto, ON M5G 2M9 Canada

**Keywords:** Network modeling, Gene regulation, Regulatory networks, Ovarian cancer, Cancer subtypes, Angiogenesis

## Abstract

**Background:**

We recently identified two robust ovarian cancer subtypes, defined by the expression of genes involved in angiogenesis, with significant differences in clinical outcome. To identify potential regulatory mechanisms that distinguish the subtypes we applied PANDA, a method that uses an integrative approach to model information flow in gene regulatory networks.

**Results:**

We find distinct differences between networks that are active in the angiogenic and non-angiogenic subtypes, largely defined by a set of key transcription factors that, although previously reported to play a role in angiogenesis, are not strongly differentially-expressed between the subtypes. Our network analysis indicates that these factors are involved in the activation (or repression) of different genes in the two subtypes, resulting in differential expression of their network targets. Mechanisms mediating differences between subtypes include a previously unrecognized pro-angiogenic role for increased genome-wide DNA methylation and complex patterns of combinatorial regulation.

**Conclusions:**

The models we develop require a shift in our interpretation of the driving factors in biological networks away from the genes themselves and toward their interactions. The observed regulatory changes between subtypes suggest therapeutic interventions that may help in the treatment of ovarian cancer.

**Electronic supplementary material:**

The online version of this article (doi:10.1186/s12859-015-0551-y) contains supplementary material, which is available to authorized users.

## Background

Ovarian cancer is the fifth leading cause of cancer death for women in the U.S. and the seventh most fatal worldwide [1]. Although ovarian cancer is notable for its initial sensitivity to platinum-based therapies, the vast majority of women eventually recur and succumb to increasingly platinum-resistant disease. Despite significant investment, improvements in patient prognosis have been slow and usually in small increments. The disease generally presents at an advanced stage (III/IV) and the five-year survival rate of advanced disease is less than 30% with median survival only slightly longer than two years [2]. Furthermore, ovarian cancer patients often undergo similar treatment regimens, mainly because the highly suspected multiple subtypes have not yet been well characterized in terms of their biological significance. In previous work, we analyzed gene expression data from 129 high-grade serous ovarian cancer samples and identified a poor-prognosis subtype characterized by the expression of angiogenic genes [3]. This subtype and the associated differences in patient survival were validated using gene expression data from a collection of 1606 ovarian cancer samples assembled from ten independent published studies. Multiple other subtypes have been proposed [4,5], but the importance of this subtyping, relative to other definitions, is that angiogenesis represents a process that is of potential clinical relevance and it is the only subtyping model which has been shown to be robust and prognostic in multiple independent datasets.

Angiogenesis is one of the well-characterized hallmarks associated with cancer progression [6], playing an important role in maintaining tumor growth [7]. Angiogenesis is facilitated by interactions between cells and the extra-cellular matrix [8,9] and is associated with increased expression among a set of particular genes, including, but not limited to, matrix metalloproteinases (MMPs) [10] and VEGF [11-13]. Angiogenesis inhibition is being intensely studied as a possible therapeutic advance in ovarian cancer, but the effects in survival are still modest, suggesting that our understanding of the molecular underpinnings and biological implications of angiogenesis in this disease is still limited [2,14]. A number of clinical trials have tested drugs targeting angiogenic factors and shown these drugs have anti-cancer activity in a subset of ovarian cancer patients [15-18]; however, better understanding of the complex mechanisms driving response to these therapies is crucial to improving their efficacy and patient outcomes [17,19].

Although we have long studied ovarian cancer from the perspective of single genes and their properties, it has become clear that more integrative, systems-level analyses are necessary to better understand how the disease and its subtypes develop and progress, and how it may respond to different therapeutic interventions. The characterization of biological processes can distinguish disease states in cases where single gene biomarkers cannot [20]. The importance of applying network approaches in particular to better understand disease has previously been highlighted [21-24]. Simultaneously, it has become evident that integrative approaches that incorporate multiple sources of data to model biological systems often yield the most informative results [25-27]. Along these lines, we recently described an integrative network inference method, PANDA (**P**assing **A**ttributes between **N**etworks for **D**ata **A**ssimilation), that models information flow in regulatory networks by searching for agreement among various data-types, using information from each to iteratively refine predictions in the others [28].

PANDA models network interactions as communication between “transmitters” and “receivers”. In the context of PANDA’s regulatory networks, the transmitters are transcription factors and the receivers are their downstream target genes. This approach recognizes that for communication to occur, both the transmitter and the receiver have an essential role – although a transcription factor is *responsible* for regulating a target gene, the gene must also be *available* to be regulated. By constructing a “prior” regulatory network consisting of potential routes for communication (for example, by mapping transcription factor motifs to a reference genome) and integrating with other sources of regulatory information (such as protein interaction and gene expression data), one can estimate the *responsibility* and *availability* of each potential interaction, predict where communication is succeeding and failing, and deduce condition-specific network structures.

Here we describe the application of PANDA to reconstructing subtype-specific regulatory networks in ovarian cancer. We identify differences in network topologies between the angiogenic and non-angiogenic subtypes, and use this information to suggest potential therapies that may be efficacious in treating patients with angiogenic-subtype ovarian tumors.

## Results and discussion

### Building angiogenic and non-angiogenic specific regulatory network modules

**Figure 1:**
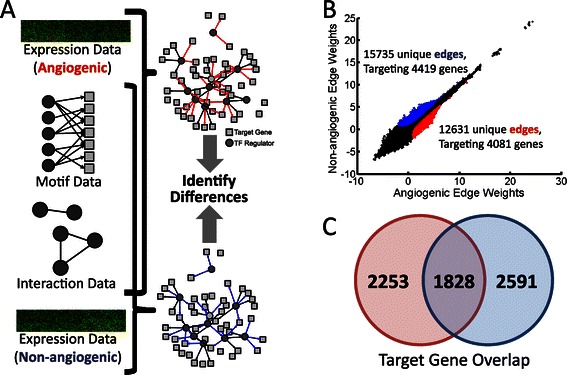
A summary of PANDA gene regulatory network reconstruction and the identification of subtype-specific subnetworks. (**A**) We combine transcription factor motif and physical protein-protein interaction (PPI) data with gene expression data from each subtype to build individual network models. (**B**) We compare the weights of edges predicted by PANDA for each of the network models. Each point in the graph represents an edge connecting a TF to a target gene. We also define subnetworks by selecting high-probability edges specific to either the angiogenic (red) and non-angiogenic (blue) model. The number of edges and genes identified as part of these subnetworks is noted. (**C**) Although the subnetworks contain unique sets of edges, genes targeted by TFs in these interactions are not necessarily unique, suggesting distinct regulatory processes.

To begin, we downloaded mRNA expression data for 510 ovarian samples profiled by the Cancer Genome Atlas (TCGA) (https://tcga-data.nci.nih.gov/tcga/tcgaHome2.jsp, [5]), normalized this data using fRMA [29], and mapped probes to Ensembl identifiers using the biomaRt Bioconductor package version 2.8.1 [30]. As reported in [3] we classified samples as belonging to either the angiogenic or non-angiogenic subtype; 188 were classified as part of the angiogenic subtype, and 322 were classified as non-angiogenic. We constructed separate genome-wide regulatory network models for the two subtypes. We began by mapping 111 TFs with known binding motifs to the promoters, here defined as [−750,+250] base-pairs around the transcription start site, of the 12290 genes with expression data in TCGA samples. Because transcriptional regulation involves assembly of protein complexes and allows for combinatorial regulatory processes, we collected information regarding physical protein interaction data between human transcription factors estimated using a mouse-2-hybrid assay [31]. We used PANDA to integrate information from transcription factor binding motifs, protein-protein interaction data, and subtype-specific gene expression, constructing directed transcriptional regulatory networks for the angiogenic and non-angiogenic ovarian cancer subtypes (Figure 1A).

For each edge that connects a transcription factor to its target gene, PANDA assigns a weight, in z-score units, that reflects the confidence level of a potential inferred regulatory relationship. Not surprisingly, we found the edge weights for the angiogenic and non-angiogenic regulatory networks to be highly correlated (Figure 1B), representing common regulatory mechanisms and processes active in both subtypes. However, we also found regulatory edges that were more strongly supported by either the angiogenic or the non-angiogenic subtype. We identified 12631 edges that we assigned to an angiogenic subnetwork, shown in red, and 15735 edges for the non-angiogenic subnetwork, shown in blue. The edges in the angiogenic subnetwork target 4081 genes while the non-angiogenic subnetwork edges target 4419; of these, 1828 genes are targeted in both subnetworks (Figure 1C), although by different upstream transcription factors. This may reflect the fact that in addition to different pathways being activated in each of the subtypes, there may also be a complex “re-wiring” of the networks around commonly targeted genes.

### Network analysis reveals biological mechanisms associated with regulatory differences

We compared the corresponding subnetworks and identified a subset of transcription factors associated with the strongest regulatory changes. To do this we identified the genes targeted for regulation by a transcription factor in each of the two subnetworks and determined both an “edge enrichment” score as well as a p-value significance for the difference in the number of target genes. Specifically, we define “edge enrichment” for each transcription factor as the out-degree of that transcription factor (number of edges pointing out to a target gene) in the angiogenic subnetwork divided by the out-degree for the same transcription factor in the non-angiogenic subnetwork (multiplied by a normalization factor equal to the total number of edges in the angiogenic subnetwork divided by the total number of edges in the non-angiogenic subnetwork); the statistical significance (quantified as a p-value) of the rewiring is determined by using the hypergeometric distribution model to evaluate the overlap between edges from a transcription factor and edges specific to a particular subnetwork.

**Figure 2:**
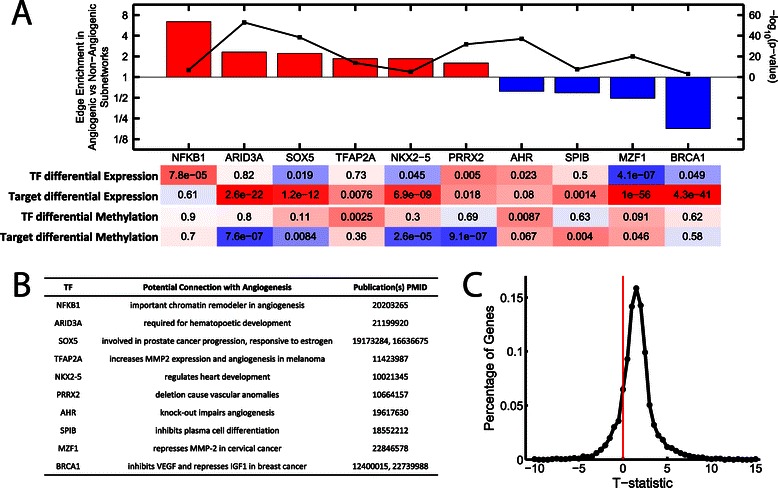
A summary of some of the key regulatory events distinguishing the two subnetworks. (**A**) Transcriptional factors that have significantly more targets in one of the subnetworks compared to the other (edge enrichment > 1.5, p < 1e-3). The differential expression and methylation of each transcription factor as well as the differential expression/methylation of its target genes (in the corresponding subnetwork) is noted. Color corresponds to direction of differential expression/methylation (red: higher in angiogenic, blue: higher in non-angiogenic). (**B**) Genetic functions associated with these key transcription factors describing their potential role in angiogenesis and ovarian cancer. (**C**) A distribution of the t-statistic for differential methylation across all the genes. The shift of the distribution to the right indicates an overall increase in methylation in the angiogenic compared to the non-angiogenic samples.

On average, a given transcription factor will be associated with around 114 edges in the subtype-specific subnetworks. However some transcription factors are associated with a relatively small number (less than twenty) of edges. These transcription factors were excluded in further analysis to enhance statistical robustness. We identified ten “key” transcription factors with an edge enrichment greater than 1.5 (or less than 1/1.5) and with p < 10^−3^ (Figure 2A). The identified transcription factors all have established associations with angiogenesis or survival ([32-43], Figure 2B). For example NFKB1 is important for chromatin remodeling during angiogenesis [32], PRRX1 deletion causes vascular anomalies [38], and MZF1 can repress MMP2 [41], a key prognostic factor in ovarian cancer [44].

We tested if these ten transcription factors would also be identified in a simple differential expression analysis using a *t*-test. Only three out of ten TFs are differentially expressed at the p < 0.01 cutoff: NFKB1 (more highly expressed in the angiogenic subtype, p = 7.8 × 10^−5^, FDR = 4.3 × 10^−4^), PRRX2 (more highly expressed in the angiogenic subtype, p = 0.005, FDR = 0.015), and MZF1 (more highly expressed in the non-angiogenic subtype, p = 4.1 × 10^−7^, FDR = 4.2 × 10^−6^). Thus, PANDA identified transcription factors that are not strongly differentially expressed between the subtypes yet are known to participate in angiogenic processes.

We also tested targets of each transcription factor for differential expression (see Methods) and found significant differences in target expression for six of the seven remaining TFs. For example, ARID3A is not differentially expressed between angiogenic and non-angiogenic subtypes, but its targets have significantly (p = 2.6 × 10^−22^) increased expression in the angiogenic subtype. This suggests that transcription factor activity changes may not be detectable based on their own expression level increases or decreases, but that the expression of their targets can provide information on how they influence phenotype.

There are many factors that could contribute to expression abnormalities in cancer, such as differences in mutations, copy-number variation and epigenetic states. We sought to determine which, if any, of these, might be contributing to the differential expression of the genes targeted by each of our ten key transcription factors. First, we investigated whether copy-number variation might explain the overall change in gene expression (see Methods). Although we find some nominally significant changes for the targets of PRRX2 (p = 0.0029) and ARID3A (p = 0.0257), these genes actually have less overall amplification in the agiogenic subtype compared to the non-angiogenic subtype (t = −2.98 and t = −2.23 respectively). This is despite the fact that their mRNA expression levels are higher in the angiogenic compared to non-angiogenic subtype. Thus copy-number variation does not appear to be the primary factor driving changes in expression of these target genes.

Epigenetic factors provide an alternative mechanism for differential targeting by these transcription factors. To explore this possibility we mapped DNA methylation data from TCGA to the samples and genes used in our network reconstruction. We used a *t*-test to quantify any potential change in DNA methylation for each gene between the angiogenic and non-angiogenic subtypes and show the distribution of these values in Figure 2C. Overall we found DNA methylation levels to be higher in angiogenic tumor samples (mean value of t-statistic across all genes is 1.52). We next determined the differential methylation of the ten transcription factors and their targets in each of the subnetworks (Figure 2A). Compared to the rest of the genome, genes targeted by four of the ten transcription factors, ARID3A, SOX5, NKX2-5 and PRRX2, are associated with significantly lower methylation in angiogenic samples. It should be noted that this lower level of methylation does not indicate hypomethylation. In fact, the average t-statistic value for the targets of these transcription factors is greater than zero (ARID3A, t = 1.17; SOX5, t = 1.31; NKX2-5, t = 0.70; PRRX2, t = 1.24), indicating that their targets, on average, have higher levels of methylation in the angiogenic compared to the non-angiogenic subtype; however, that increase in methylation is comparatively less than that experienced by genes not targeted by these regulators.

We note that the TCGA methylation data was not used by PANDA to construct the networks, yet is highly concordant with the predicted patterns of gene regulation. Although many of the transcription factors that alter targeting between the aniogenic and non-angiogenic subnetworks are not significantly differentially-expressed between the subnetworks, their targets genes often are. At the same time, these target genes are also differentially-methylated between the subtypes. Overall this analysis provides independent support of the overall network model.

### Both transcriptional activation and repression are used to control angiogenic pathways

Transcription factors can either activate or repress gene expression. The target gene expression analysis in Figure 2A provides a preliminary indication about potential regulatory roles for the identified transcription factors. For example, although MZF1 and BRCA1 exhibit an edge-enrichment in the non-angiogenic subnetwork and are themselves also more highly expressed in the non-angiogenic samples, their *targets* show the opposite trend, with significantly higher expression in the angiogenic samples (p = 1.0 × 10^−56^ and 4.3 × 10^−41^, respectively). There are two scenarios consistent with these observations: (1) loss of control by these transcription factors results in the increased expression of their former targets, and (2) increased control by these transcription factors results in the decreased expression of their target genes. Interestingly, BRCA1 is known to negatively regulate IGF1 expression in breast cancer cells [43], which could inhibit angiogenesis as multiple studies have shown that increased levels of IGF1 in cancer calls leads to an increase in cell proliferation [45-47]. Similarly, MZF1 is a repressor of MMP2 [41] and is known to inhibit hematopoietic lineage differentiation in embryonic stem cells [48]. Combined with the analysis presented in Figure 2A, this suggests that MZF1 and BRCA1 act as transcriptional repressors in the non-angiogenic samples.

**Figure 3:**
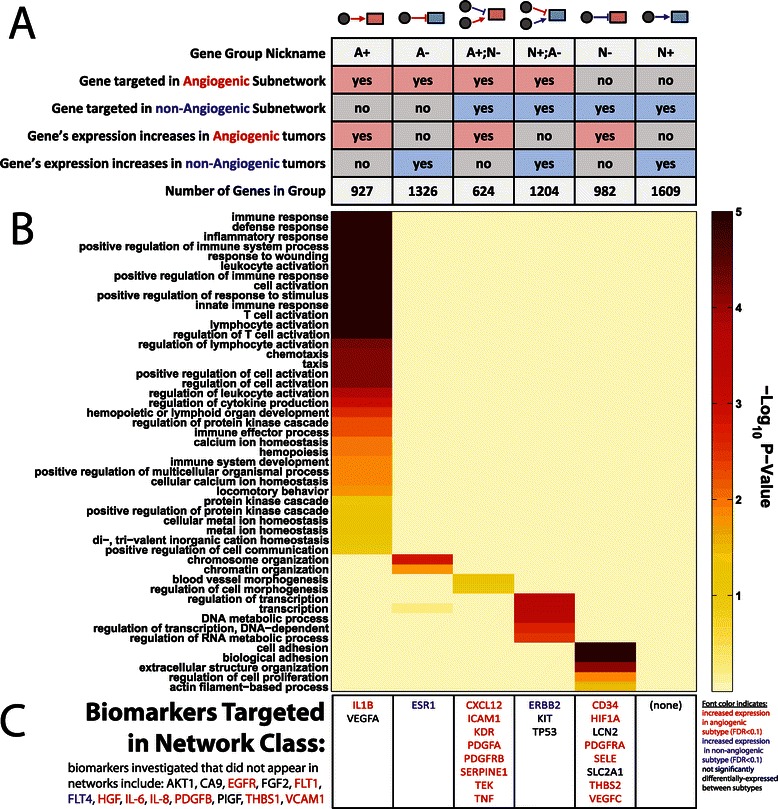
Characteristics of six classes of differentially-targeted genes. (**A**) The classification of genes based on whether evidence suggests that the regulatory interactions targeting those genes are activating, repressive, or both. (**B**) Enriched Biological Process Gene Ontology terms (FDR < 0.1) associated with at least one of these six classes; we only included categories with at least 100 gene annotations. FDR significance is shown as a color with darker colors representing more significant enrichment. (**C**) Potential angiogenesis biomarkers that belong to each of the six classes of genes. Biomarkers differentially-expressed between the subtypes at an FDR < 0.1 are noted in red or blue based on whether they are more highly expressed in the angiogenic or non-angiogenic subtypes, respectively.

Motivated by these observations, we classified the edges in our two subnetworks as either “activating” or “repressing” based on whether changes in the target gene's expression is correlated or anti-correlated with subnetwork assignment. We then assigned each target gene to one of six non-overlapping classes (see Figure 3A):

1. “A+”: genes targeted only in the angiogenic subnetwork that are more highly expressed in the angiogenic subtype;
2. “A-”: genes targeted only in the angiogenic subnetwork that are more highly expressed in the non-angiogenic subtype;
3. “A+;N-”: genes targeted in both subnetworks and more highly expressed in the angiogenic subtype;
4. “N+;A-”: genes targeted in both subnetworks and more highly expressed in the non-angiogenic subtype;
5. “N-”: genes targeted only in the non-angiogenic subnetwork that are more highly expressed in the angiogenic subtype;
6. “N+”: genes targeted only in the non-angiogenic subnetwork that are more highly expressed in the non-angiogenic subtype.

We used DAVID [49] to test for functional enrichment in these six classes of target genes, with the 12290 network genes taken as a background. The FDR p-values for GO “Biological Process” categories with more than one hundred members that have an FDR enrichment of less than 0.1 in at least one of our six classes of genes are illustrated as a heat map in Figure 3B.

The angiogenic-activated class (“A+”) has the greatest number of significantly enriched functional categories. Many of these are associated with immune-response; processes associated with angiogenesis are also included, for example “chemotaxis,” “hematopoiesis,” “positive regulation of cell communication” and “metal ion homeostasis.” Some processes found to be enriched for genes repressed in the non-angiogenic subnetwork (“N-” genes), such as “cell adhesion” and “extracellular structure organization,” also play a role in angiogenesis. In addition, genes activated in the angiogenic subnetwork but repressed in the non-angiogenic subnetwork (“A+;N-”) include those involved in “blood vessel morphogenesis.” This suggests angiogenesis involves not only the activation of certain genes in the angiogenic subtype, but also removal of repressive regulatory interactions from the non-angiogenic subtype. In contrast, genes repressed in the angiogenic subnetwork (“A-” genes) are associated with “chromatin organization,” consistent with the observed role that epigenetics plays in distinguishing the subtypes (see Figure 2C). Genes activated in the non-angiogenic subnetwork but repressed in the angiogenic-subnetwork (“N+;A-” genes) are involved in functions such as “transcription” and “DNA metabolic process”.

We also investigated whether previously identified potential biomarkers for angiogenesis were targeted in our networks, and, if so, which “class” of genes those biomarkers belonged to. In particular, we investigated thirty-five biomarkers, described in [50,51], and found twenty-two targeted in our defined subnetworks (Figure 3C). The majority (eighteen) of these biomarkers are targeted in either the “A+”, “A+;N-“or “N-“class, consistent with higher expression in the angiogenic subtype. Interestingly, many of these biomarkers are targeted in the *non-angiogenic* subnetwork (“A+;N-“or “N-“classes). One possible interpretation of these results is that *repressive* regulatory features play a role in inhibiting angiogenic progression, in addition to transcriptional activation of these biomarkers in angiogenic tumors. Curiously, three of the four biomarkers not included in the pro-angiogenic network classes were identified in only a single study [52] that included twenty patients with inflammatory breast cancer [50]. We note that while many of the network-targeted biomarkers are also significantly differentially-expressed between the subtypes (FDR < 0.1 based on un-paired *t*-test), several are not, including VEGFA (FDR = 0.69), TP53 (FDR = 0.12), LCN2 (FDR = 0.68), KIT (FDR = 0.73) and SLC2A1 (FDR = 0.10). The identification of VEGFA and other biomarkers in our network model despite clear lack of differential-expression may indicate that our network model is able to identify important cellular regulatory alterations even in the absence of distinct changes in downstream target gene expression.

### Combinatorial control plays a critical role in potentiating angiogenesis

**Figure 4:**
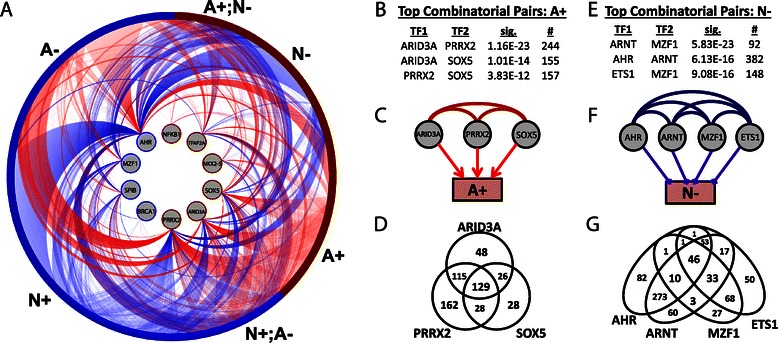
Characterizing combinatorial regulation in the subnetworks. (**A**) An illustration of the identified key active subnetworks. Identified key transcription factors form the inner ring and their target genes the outer ring. Target genes are colored based on whether they are more highly expressed in the angiogenic (red) or non-angiogenic subtype (blue) and are organized based on their classification. Angiogenic subnetwork edges (red) and non-angiogenic subnetwork edges (blue) extend between these rings, from the regulating transcription factor in the inner ring, to its target gene in the outer ring. (**B**) A table of the top three co-regulatory TF-pairs targeting “A+” genes, (**C**) a diagram illustrating these co-regulatory interactions, and (**D**) a Venn-diagram showing the overlap of the “A+” genes targeted by these TFs. (**E**) A table of the top three co-regulatory TF-pairs targeting “N-” genes, (**F**) a diagram illustrating all significant co-regulatory events between these TFs in “N-” genes, and (**G**) a Venn diagram showing the overlap of “N-” genes targeted by each of these TFs.

Regulatory information that pertains to the core of our network can be depicted using a ring diagram representing the union of the two subnetworks (Figure 4A). In this visualization, our ten key regulators form the inner ring, while their targets, colored based on whether they exhibit higher average expression in the angiogenic (red) or non-angiogenic (blue) samples, form the outer ring. Viewing the two subnetworks, it is clear that there is a high degree of combinatorial gene regulation. To quantify this, we applied the hypergeometric distribution model and, using the union of the genes targeted in both the angiogeneic and non-angiogenic networks as a background, tested for over-representation of genes co-targeted by specific pairs of transcription factors in the various network classifications (either “A+,” “A-,” “N+” and “N-”). Here, we focus on the three most significant pairs that include at least one of the ten identified key transcription factors. Information for all pairs can be found in the Supplemental Material (Additional file 1: Dataset S1). Note that the genes in the “A-” and “N+” classes had no combinatorial pairs significantly enriched (using a p = 10^−3^ cutoff).

For “A+” genes, as was seen in Figure 4A, we identify significant co-regulatory associations between ARID3A, PRRX2, and SOX5 (Figure 4B-C) and these three regulators share many common targets (Figure 4D). In fact, 58% of the “A+” genes are targeted by at least one of these transcription factors, 32% by at least two, and 14% are targeted by all three, suggesting they may function as a module that coordinately regulates these genes.

For the “N-” genes, the top three significant co-regulatory transcription factor pairs include combinations of ETS1, ARNT (also known as HIF1β), MZF1 and AHR (Figure 4E). All possible pairs of these four TFs (including those that don’t include one of our “key” transcription factors) are enriched in “N-” genes at a statistically significant level (p < 1 × 10^−6^, Figure 4F), although MZF1 generally only shares targets of AHR or ARNT in combination with ETS1 (Figure 4G). AHR and MZF1 are among our key regulators, and, as noted previously, MZF1 is known to repress MMP2 and reduced cancer invasiveness [41]. However, ETS1 and ARNT were not among our list of key regulators, indicating that combinatorial events might be especially important for these two transcription factors. Previous reports suggest that although various ETS family members can either activate or repress angiogenic pathways [53,54], ETS1, in particular, acts as a mediator of angiogenesis [55], dimerizing with HIF2α to *activate* VEGFR1 and VEGFR2 [56,57]. Similarly, ARNT dimerizes with HIF1α to activate VEGF and angiogenesis [58]. However, the dimerization of *AHR* with ARNT *inhibits* ARNT/HIF1α dimerization, thereby reducing VEGF production and subsequent angiogenesis [59]. Thus, even though ARNT/HIF1α promotes angiogenesis, the fact that *ARNT/AHR* dimerization *inhibits* angiogenesis offers an explanation for our observation that ARNT is associated with the repression of genes. Since both ETS1/HIF2α and ARNT/HIF1α interactions occur through a PAS domain [60,61], it is likely that a similar mechanism underlies our observed combinatorial enrichment of ETS1 with AHR and we hypothesize that ETS1 interaction with AHR prevents dimerization with HIFα proteins, thereby reducing VEGF production and subsequent angiogenesis.

### The network model captures the effects of various treatment strategies

**Figure 5:**
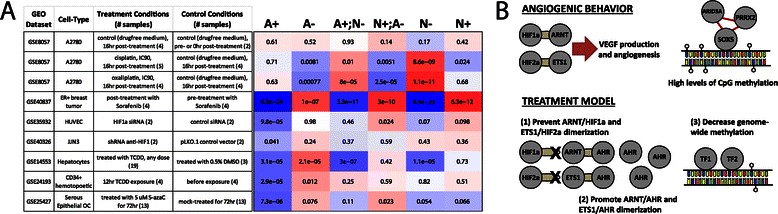
Proposed therapeutic approaches. (**A**) A summary of the results found by comparing the expression patterns of genes in “treatment” versus “control” samples in each of the GEO datasets to “classes” of genes defined in our network analysis. We report the significance of association of differential expression with the indicated gene class, using a GSEA approach [110]; colors indicate direction of differential expression (red - increase upon treatment, blue - decrease upon treatment). (**B**) An illustration of some of the key findings regarding the potential mechanisms driving angiogenesis in ovarian cancer found using PANDA, as well as three potential treatments that may inhibit angiogenesis.

We wished to investigate how genes identified using our network model might respond to standard or other treatment protocols. Therefore, we analyzed experimental data (GEO accession numbers GSE8057, GSE40837) measuring gene expression levels in response to several chemotherapy drugs that are commonly used to treat ovarian cancer patients and/or angiogenesis, including cisplatin, oxaliplatin and sorafenib. For each experiment, we used RMA [62] to normalize gene expression CEL files downloaded from the Gene Expression Omnibus and used a custom-CDF to map to Entrez GeneIDs [63]. We selected samples that correspond to either a treatment or control experiment and performed a *t*-test to quantify the differential expression of all genes between these sets of samples. Finally, we computed a summary statistic representing the aggregate differential expression value for the sets of genes within each of the six “classes” defined by our subnetworks (for more details, see Methods). The results are summarized in Figure 5A; intensity of red or blue coloration scales with the significance of increased or decreased expression, respectively, in the treatment compared to the control samples, for the genes belonging to each of our networks “classes”.

Platinum-based therapies are widely used in ovarian cancer treatment regimens. Therefore, we began by investigating the effect of the chemotherapy drugs cisplatin and oxaliplatin on the expression levels of genes in A2780 human ovarian carcinoma cells (GSE8057, [64]). As a negative control, we compared expression levels of cells grown in a drug-free medium for 16 hours to their expression at 0 hours. As expected, there is little differential expression and we observe no association with any of our classes of genes (Figure 5A). We next compared expression of cells grown for 16 hours following treatment with either cisplatin or oxaliplatin to those grown for 16 hours in drug-free medium. Curiously, in both instances we see that genes normally repressed in the non-angiogenic subnetwork (“N-”) actually increase their expression levels following treatment, suggesting that these drugs disrupt regulatory interactions that are important for *repressing* angiogenic activities. This is consistent with the results of a previous study [65] showing that when taken in isolation, cisplatin is not effective for treating angiogenesis in ovarian cancer.

Although decreased expression in “A+” genes does not occur following platinum-based treatment, we reasoned that the effects of a VEGF-inhibiting drug should be reflected in our identified subnetworks. Biopsies of ER+ breast tumors have been collected from patients both prior to and following a clinical trial of sorafenib, and the genome-wide expression levels of genes were measured in these samples (GSE40837). Analysis of this data in the same manner as the platinum-based therapies shows a striking association with the subnetworks (Figure 5A). Genes in the “A+,” “A+;N-” and “N-” groups all show a profound decrease in expression post-treatment while genes in the “A-,” “A-;N+” and “N+” groups all increase their expression. Although this is breast rather than ovarian cancer, these results are exciting since they rely on patient samples collected from a clinical trial rather than cell-lines, illustrating a patient-level association of an angiogenic-inhibition drug among network-defined genes. This result also serves as a positive control on our network analysis. We note that the six classes of genes we define are not wholey independent of the gene expression data, which we used both to reconstruct the networks as well as to divide target genes into distinct classes. Consequently, we would expect similar results for analyses of other “classes” of genes whose differential-expression is associated with differences between the two subtypes. However, analyzing the networks has the potential to provide additional mechanistic insight into differences between the subtypes and identify other drugs not classically associated with angiogenesis.

### Three treatments may synergistically inhibit angiogenic progression

The optimal angiogenesis-based treatment in ovarian cancer is still a matter of ongoing investigation [14]. Commonly-used anti-angiogenic drugs mainly target VEGF, a major contributor to angiogenesis. On the other hand, as described below and illustrated in Figure 5B, several mechanisms highlighted in our network analysis suggest alternate approaches for treatment that, although speculative, could utilize existing compounds to control, or potentially reverse, angiogenesis in ovarian cancer. For each of these proposed treatments we identified highly-related compounds and ascertained if there is a verifiable effect on gene expression in either ovarian cancer or another human system.

#### ARNT and ETS1 dimerization with HIF1α and HIF2α, respectively, must be prevented

As noted above, the dimerization of ETS1 and ARNT with HIF1α and HIF2α, respectively, generally promotes angiogenesis, although AHR may be interfering to repress target gene expression in the non-angiogenic subtype. It is therefore essential that the dimerization of these HIF proteins with ARNT and ETS1 is inhibited. HIF2α dimerizes with ARNT through a PAS-B domain, located on the C-terminus of the ARNT protein. The structural basis for this dimerization has been solved [66,67] and a small molecule ligand has been identified that dimerizes with HIF2α, decreasing affinity of the ARNT/HIF2α heterodimer [60]. Similarly, a compound has been identified that dimerizes with HIF1α, decreasing the affinity of the ARNT/HIF1α heterodimer [68]. Using either or both of these compounds, we believe one could prevent or reverse angiogenic effects driven by ARNT/HIF1α and perhaps also those driven by ETS1/HIF2α dimerization. In lieu of a dimerization inhibitor, we investigated how siRNA depletion of HIF1α affects the expression levels of genes in our identified subnetworks. We observe that in two independent experiments [69,70], “A+” genes exhibit a decrease in expression upon HIFα depletion, as we would expect from our model (Figure 5A).

#### AHR dimerization with ARNT and ETS1 must be promoted

Preventing the dimerization of ARNT/HIF1α and ETS1/HIF2α may be insufficient; inhibition of angiogenesis is also contingent upon the dimerization of ARNT with AHR (and perhaps also ETS1/AHR). Consequently, we also suggest treatment with an AHR agonist, such as the selective AHR modular (SAhRM) 6-methyl-1,3,8-trichlorodibensofuran (6-MCDF), which has been shown to inhibit carcinogen-induced mammary tumor growth in rats [71]. TRAMP mice fed 6-MCDF in their diet had overall lower levels of serum VEFG and were five times less likely to have metastasis compared to mice on a control diet [72]. Although a known carcinogen, one of the most efficient AHR agonists is the environmental toxin 2,3,7,8-Tetrachlorodibenzo-p-dioxin (TCDD). TCCD has been shown to potentiate ARNT/AHR dimerization thereby inhibiting angiogenesis and preventing vascular remodeling in rat placenta [73]. Curiously, accidental exposure to TCCD was found to potentially decrease incidence of breast and endometrial cancer in a group of women [74]. In our network, “A+” genes show a decrease in expression upon treatment with the AHR agonist TCDD in both hepatocytes [75] and CD43+ hematopoetic cells [76] (Figure 5A). The hepatocyte data also shows a significant decrease in expression for “A+;N-” genes and “N-” genes, and an increase in expression for “A-” genes.

#### Methylation levels across the entire angiogenic genome must be decreased

In patients whose tumors have already become angiogenic, epigenetic alterations may need to be considered. One hallmark of many cancers is alteration of DNA methylation and, indeed, we found higher genome-wide methylation levels in the angiogenic subtype (Figure 2C). Interestingly, SOX5, one of our “key” transcription factors that was also found to play an important combinatorial role with ARID3A and PRRX2, contains an HMG-box which binds the minor groove [77]. Since methylation modifications occupy the major groove of DNA [78], this implies that SOX5 might be mediating key regulatory processes in the angiogenic subtype in a methylation-independent manner. This suggests that it may be necessary to decrease methylation levels across the angiogenic genome, thereby increasing competition with SOX5 binding to gene promoters, and altering their subsequent expression. This could be achieved using, for example, DNA methyltransferase (DNMT) and histone deacetylase (HDAC) inhibitors, which have already shown potential to inhibit angiogenesis in other systems [79,80]; such treatments in ovarian cancer might yield similar results. A hypomethylating agent, such as 5-azacytedine, could also be used to alter the epigenetic landscape and control angiogenic progression. With this in mind, we investigated the expression of ovarian cancer cells both prior to and post treatment with 5-azacytedine [81]; we observe a decrease in expression of the “A+” genes, consistent with our hypotheses (Figure 5A).

### Additional potential therapies associated with differentially-targeted genes

We have identified several treatment options that target specific biological mechanisms uncovered when contrasting our network models; however, these are the result of intensive literature mining to determine suitable candidate drugs. We used the connectivity map (CMAP) [82] to determine if a gene classification based on our network analysis could also be used to identify potential drugs for treating angiogenesis in ovarian cancer.

We first used genes assigned to the “A+” and “A-” classification (see Figure 3) to build a “network-signature” suitable for CMAP analysis. As expression information was included in our network model, we also built an “expression-signature” by selecting genes with the most significant changes in expression between in the angiogenic and the non-angiogenic subtypes (based on an unpaired *t*-test). A cutoff of p < 1.6e-6 (FDR < 1.45 × 10^−5^) was used to select genes more highly expressed in the angiogenic subtype and p < 3.05e-4 (FDR < 1.4 × 10^−3^) to select genes more highly expressed in the non-angiogenic subtype. We used these criteria to select differentially-expressed genes so that the expression-signature and network-signature had equivalent dimensions (920 “up” genes and 1287 “down” genes in both cases). These two signatures share approximately 20% of their genes, with 225 in common in the “up” direction and 223 in common in the “down” direction. We used CMAP to identify drugs associated with each of these signatures. Comparing results from these two signatures will allow us to distinguish between drug candidates only identified in the network context, and those which would also be identified using a differential-expression analysis.

Table 1 lists drugs significantly associated with the network-signature classification (p < 0.01). Most of these drugs are also significantly associated with the expression-signature -- not surprising given that these two signatures are non-independent; however, the ranking from the expression-signature is vastly different from that of the network-signature. One significant exception is Prestwick-675, or hippeastrine, which is ranked highly in both analyses. Hippeastrine is an amaryllidaceae alkaloid with potent anti-invasive properties [83] and anticancer activities in cell lines [84]; it is also believed to contribute to the reported anti-cancer activities of the Chinese herb *Lycoris aurea* [85].

**Table 1:** **The drugs significantly associated (FDR < 0.1) with the network-signature classification based on CMAP analysis**

**Drug name**	**CMAP rank**		**CMAP P-value**		**CMAP FDR**	
	**Network signature**	**Expression signature**	**Network signature**	**Expression signature**	**Network signature**	**Expression signature**
Harmol	1	61	4.6E-04	1.1 × 10^−3^	0.015	4.9 × 10^−3^
Harmalol	2	67	4.9E-04	1.8 × 10^−3^	0.015	7.1 × 10^−3^
Brinzolamide	3	95	5.6E-04	5.8 × 10^−3^	0.016	0.020
Edrophonium chloride	4	42	6.0E-04	3.6 × 10^−4^	0.017	1.8 × 10^−3^
Trimethadione	5	138	6.8E-04	0.035	0.018	0.101
Canavanine	6	39	7.0E-04	2.7 × 10^−4^	0.018	1.5 × 10^−3^
Oxamniquine	7	49	1.3E-03	6.0 × 10^−4^	0.025	2.8 × 10^−3^
Metolazone	8	59	1.3E-03	1.0 × 10^−3^	0.025	4.4 × 10^−3^
Etofylline	9	112	3.2E-03	0.015	0.048	0.048
Harmine**	**10**	**613**	**3.6E-03**	**1.000**	**0.051**	**1.000**
Tetracycline	11	108	4.0E-03	0.013	0.056	0.041
Sotalol	12	136	5.5E-03	0.033	0.069	0.096
5213008	13	55	6.5E-03	8.1 × 10^−4^	0.080	3.7 × 10^−3^
Prestwick-675	14	1	7.3E-03	6.0 × 10^−6^	0.084	5.2 × 10^−5^
Pirinixic acid	15	21	8.6E-03	2.4 × 10^−5^	0.095	1.8 × 10^−4^
Dicoumarol**	**16**	**184**	**8.8E-03**	**0.239**	**0.097**	**0.534**
Pentoxyverine**	**17**	**784**	**9.1E-03**	**1.000**	**0.098**	**1.000**
Adipiodone**	**18**	**689**	**0.010**	**1.000**	**0.100**	**1.000**

Drugs that are not significantly associated with the expression-signature are bolded and asterisked (**).

One of the most striking results from this analysis is that several drugs are significantly associated with the network-signature but not the expression-signature. The antitussive pentoxyverine is an agonist of the sigma-1 receptor [86], which has been shown to contribute to the induction of cancer-specific apoptosis by interleukin-24, a known inhibitor of angiogenesis [87]. Dicoumarol is an anticoagulant that is often administered to cancer patients. Anticoagulants are believed to be able to interfere with tumor angiogenesis [88] and in clinical trials their overall association with improved patient-survival, while encouraging, may to be limited to a subset of cancers [89]. Dicoumarol, in particular, inhibits furin-like activity by blocking the processing of MMP1 [34] and has been shown to abolish the TNF-induced activation of NFKB1 [90], one of our identified “core” transcription factors.

Another interesting drug identified by the network-signature is harmine. Harmine was recently shown to suppress tumor growth by inhibiting angiogenic activities in endothelial cells [91] and to induce apoptosis by inhibiting the expression of MMP2 in gastric cancer [92]. Interestingly, in light of the network model we propose above, harman, a related alkaloid, stimulates AHR-dependent luciferase activity [93], and harmine is a competing ligand with the well-known AHR-agonist TCDD [94]. The association of these alkaloids with the network-signature is strengthened by the fact that two of harmine’s sister compounds, harmol and harmalol, were identified as the top compounds most significantly associated with the network-signature.

## Conclusions

Although a wealth of cancer gene expression data has been generated over the last decade, most biological inference has been based on statistical tests at the level of individual genes (with very high rates of spurious associations) followed by functional meta-analysis using gene set enrichment. Our network analysis of angiogenic and non-angiogenic phenotypes in ovarian cancer led us not just to differential expression, but also to the underlying regulatory mechanisms associated with the differential activity of transcriptional programs. By associating differences in regulatory patterns with differences in gene expression we were able to define subsets of genes that are activated or repressed by their regulators. Then, by identifying and exploring relationships between a set of key transcriptional regulators, we were able to identify putative mechanisms by which they might be coordinately working together to activate, or repress, the expression of their target genes. Based on these observations we propose three therapeutic strategies that may complement or replace currently-used anti-angiogenic treatments. While these proposed strategies remain speculative, and experimental validation will be critical in validating their efficacy, the strategies are supported by our analysis of experiments from independent, published gene expression datasets, where mechanisms closely relevant to those predicted by our models were tested. We anticipate that these treatments could be combined synergistically to better inhibit angiogenesis in ovarian cancer tumors and bypass resistance that develops with the use mono-therapeutic conventional angiogenesis inhibitor regimens.

Until recently no clinical trials have collected the expression data needed to validate the angiogenic subtype classification in ovarian cancer, and consequently there is currently no experimental data supporting increased angiogenesis in the angiogenic subtype. However, there is strong anecdotal evidence supporting the classification, including response rates consistent with what we would have predicted in clinical trials involving angiogenesis inhibitors [95-97]. The use of PANDA in comparing the subtypes led to an independent identification of angiogenic processes, among others. Importantly, PANDA itself does not rely on differential-expression, but rather characterizes the targeting of genes by transcription factors. Indeed, we found that many of the transcription factors with the greatest differential-targeting between the subtype-specific subnetworks are not themselves strongly differentially-expressed between the two subtypes. However, subsequent analysis led us to identify potential therapies that could disrupt the processes that distinguish the subtypes, which are only coincidentally associated with angiogenesis.

The clinical impact of current anti-angiogneic therapies on the outcome of ovarian cancer and other cancers, although real, continues to be modest, despite early highly promising results in mouse models [98]. In seminal ovarian cancer clinical trials only a small amount of improvement in progression free survival (four months) was observed with bevacizumab treatment [99] and angiogenesis inhibitor resistance is frequently seen *de novo*, or over the course of treatment [100]. In addition, several attempts to define biomarkers of clinical response to anti-angiogenesis drugs, have failed to produce a singular strong or consistently predictive biomarker [50,51,101-104]. Such therapeutic and predictive limitations may also reflect our limited understanding of the specific underlying mechanisms driving angiogenic progression. Indeed, we observe that the activity of many previously identified potential biomarkers for angiogenesis may be modulated through complex regulatory features (see Figure 3C) that include an important role both for coordinated transcriptional activation and repression. For instance, our analysis revealed that VEGFA is targeted in the angiogenic network but not differentially expressed between the subtypes. Furthermore, markers such as HIF1 (a proangiogenic factor) and PDGFRA (a kinase contributing to angiogeneisis and frequently targeted by angiogenesis inhibitor drugs), while having increased expression in the angiogenic tumor subtype, were identified as targeted for repression in the non-angiogenic subnetwork. Thus, our proposed network and treatment models begin to address, in more depth, the complex regulatory mechanisms relevant to angiogenesis in ovarian cancer, laying the ground for a network-based subtype categorization that may allow better prediction as well as more rational therapeutic development. Importantly, the methods we use in our analyses are generalizable and could be applied to many other disease settings to suggest new therapeutic approaches.

The specific transcriptional programs activated in angiogenic ovarian cancer, and identified through our use of PANDA, underscore the complex nature of regulatory processes and point to specific interventions that may have an increased likelihood of success. While a great deal of work would be required to validate these drug candidates, and to test whether they are subtype specific, their identification and the plausibility of their specific mode of action suggest that the type of network analysis we performed can identify candidates not found through the more widely-used gene-by-gene methods for expression analysis. In diseases such as ovarian cancer, where the outcome is poor and there are few viable drug candidates, network-based methods could represent a valuable addition to the existing repertoire of tools for analyzing genomic data.

## Methods

### Constructing regulatory networks with PANDA

PANDA [28,105] uses three inputs: a motif prior, a set of known protein-protein interactions, and expression data. To create angiogenic and non-angiogenic subtype-specific transcriptional regulatory networks, we ran PANDA twice using the same transcription factor motif prior and protein-protein interaction data, but with gene expression data unique to either the angiogenic or non-angiogenic ovarian cancer subtypes. In both runs the update parameter (α) was set equal to 0.25.

#### Expression data

Gene expression data were downloaded from TCGA (https://tcga-data.nci.nih.gov/tcga/tcgaHome2.jsp), normalized using fRMA, and individual ovarian cancer samples were assigned to either the angiogenic or non-angiogenic subtype, as described in [3]. Briefly, a Gaussian mixture model was fit to the distribution of these patient scores using Mclust R package version 3.4.10 [106] and the maximum posterior probability was used to classify each sample. Of the 510 samples, 188 were classified as angiogenic, and 322 were classified as non-angiogenic. An R-package containing the data used in this manuscript has been deposited at the URL: http://bcb.dfci.harvard.edu/ovariancancer/.

#### Motif data

To create our motif prior, we downloaded the position weight matrixes (PWM) of 130 core vertebrate transcription factor binding site motifs from the JASPAR database [107,108] as processed as described in [109]. Namely, to search for motif target candidates, the motif score of each candidate S was defined as motif score = log [P(S|M)/P(S|B)], where P(S|M) is the probability to observe sequence S given the motif M, and P(S|B) is the probability to observe sequence S given the genome background B. To define motif targets, we modeled the motif score distribution by randomly sampling the genome 10^6^ times. Targets of motifs were then defined as those with a score at a significance level of p < 10^−5^. We associated genes with these motif targets if that target fell within its promoter region ([−750, 250] base-pairs around a transcriptional start site). It is possible for a motif to correspond to multiple transcription factors; in these cases we included all corresponding transcription factors. This resulted in a transcription factor to target gene mapping. From this mapping we excluded edges connected to transcription factors or genes for which we did not have expression data. This left us with a prior network from 111 transcription factors to 12290 genes.

#### Protein-protein interactions

Predicted human transcription factor interactions were obtained from [31]. We filtered these interactions to only include those between the 111 transcription factors in our motif prior and used these interactions in constructing the regulatory networks.

### Network quality estimation

To evaluate the robustness of our predicted networks we performed a number of variations on the input data used in the reconstruction and determined how it might influence the resulting estimated edge weights. The analysis shown in Additional file 2: Figure S1-S3 demonstrates the predicted networks’ robustness to jackknifing the prior edges in the motif data, the protein-protein interaction dataset used, and the samples used to estimate the two subtype networks. See the Additional file 2: Figure S1-S3 legends for more details.

### Quantitative network comparison

PANDA estimates a probability that an edge exists in an individual network and reports that estimate in terms of z-score units. We wanted to identify potential regulatory interactions that best characterized each of the subtype-specific networks. Therefore, we selected edges based both on the probability that they are “supported” in the network inference, and on whether they are “different” between the subtypes. To determine the probability that an edge is “supported,” we took the value of the inverse cumulative distribution function of a normal distribution to assign a probability value between zero and one for each edge (instead of a z-score). To determine the probability that an edge is “different” between the networks, we first subtracted the z-score weight values estimated by PANDA for the two networks and then determined the value of the inverse cumulative distribution for this difference. The product of these two probabilities represents the probability than an edge is both “supported” and “different.” We select edges for which this combined probability is greater than 80%, or:

$$ \mathrm{Edge}\ \mathrm{identified}\kern0.5em \mathrm{a}\mathrm{s}\ \left\{\left.\underline{\begin{array}{cc}\hfill angiogenic- specific\hfill & \hfill CD{F}^{-1}\left({W}_{ij}^{(A)}\right)\times CD{F}^{-1}\left({W}_{ij}^{(A)}-{W}_{ij}^{(N)}\right)>0.8\hfill \\ {}\hfill non- angiogenic- specific\hfill & \hfill CD{F}^{-1}\left({W}_{ij}^{(N)}\right)\times CD{F}^{-1}\left({W}_{ij}^{(N)}-{W}_{ij}^{(A)}\right)>0.8\hfill \\ {}\hfill neither\hfill & \hfill otherwise\hfill \end{array}}\right|\right. $$

This 80% cutoff was chosen so that each subnetwork contains roughly 1% of all possible edges. We verified the robustness of our network analysis to this cutoff by varying it systematically between 65% and 95% (see Additional file 2: Figure S4). A file with the edge-enrichment analysis for TFs performed across each of these cutoffs is also supplied in the supplemental material (Additional file 3: Dataset S2).

We recognize that there could be hidden dependencies between the z-scores so this analysis may be over-estimating the significance.

### Edge enrichment

To identify key transcription factors we calculated two values, an “edge enrichment” score as well as a p-value significance for the difference in the number of target genes. The edge enrichment for a given transcription factor (*E*_*TF*_) can be formulaically defined as: *E*_*TF*_ *= (k*_*A*_ */k*_*N*_ *)/(n*_*A*_ */n*_*N*_ *)*, where *k*_*A*_ and *k*_*N*_ are the out-degree of the TF in the angiogenic and non-angiogenic subnetwork, respectively, and *n*_*A*_ and *n*_*N*_ are the total number of edges in the angiogenic and non-angiogenic subnetworks, respectively.

The p-value significance in the overlap between edges from a transcription factor and edges specific to the angiogenic subnetwork, as modeled by the hypergeometric distribution, can then be defined as:

$$ {p}_{TF}^{(A)}={\displaystyle \sum_{k_A}^{n_A}\frac{\left(\begin{array}{c}\hfill {n}_A\hfill \\ {}\hfill {k}_A\hfill \end{array}\right)\left(\begin{array}{c}\hfill {n}_N\hfill \\ {}\hfill {k}_N\hfill \end{array}\right)}{\left(\begin{array}{c}\hfill {n}_A+{n}_N\hfill \\ {}\hfill {k}_A+{k}_N\hfill \end{array}\right)}} $$

An equivalent formula is used to calculate ${p}_{TF}^{(N)}$. For simplicity we report ${p}_{TF}^{(A)}$ for *E*_*TF*_ *> 1* and ${p}_{TF}^{(N)}$ for *E*_*TF*_ *< 1*.

In selecting “key” transcription factors we used an edge enrichment of greater than 1.5 (or less than 1/1.5), and a p-value significance less than 10^−3^. To help account for the fact that these measures are not highly robust for small out-degree values, we also only limited our analysis to transcription factors with twenty or more total edges (*k*_*A*_ *+ k*_*N*_ *≥ 20*). This last threshold resulted in excluding one potential TF, ELK4, from being identified as a “key” transcription factor. For a full list of every TF’s edge-enrichment, p-value significance and total edge count across a variety of potential subnetwork definitions, see supplemental material (Additional file 3: Dataset S2).

### Characterizing differential expression/methylation of transcription factors and their target genes

We determined the differential expression between the subtypes for each gene in our network by using the *t*-test. We determined the corresponding significance and adjusted for multiple-hypotheses by applying the Benjamini-Hochberg correction. The differential expression patterns of a set of target genes was determined by comparing the values of the t-statistic for that set of genes to the values of the t-statistic for all other genes [110]. For the same 510 samples for which we have expression data, we also downloaded level-3 methylation data from the TCGA website (https://tcga-data.nci.nih.gov/tcga/tcgaHome2.jsp) Of the 14473 genes with methylation data, we limited ourselves to the 10108 included in the expression data, 621 of which had empty values reported across all 510 patient samples analyzed. For the remaining 9487 genes, we compared methylation levels between the two subtypes using a *t*-test. We further tested for the differential methylation patterns for sets of target genes as we did for the gene expression data. Values for the differential-expression and differential methylation of each gene are included in Additional file 4: Dataset S3.

For sets of target genes we also performed a randomization procedure to ensure that the results observed in the above analysis is not coincidental (Additional file 2: Figure S5). See the supplemental figure legend for more details.

### Characterizing differential CNV for target genes

To evaluate changes in copy-number for sets of target genes, we downloaded level 3 CNV (SNP Array) data files from TGCA. According to the TCGA documentation, these files contain the results of CBS segmentation of the log R ratio data for each tumor/normal pair. We identified all the segments in which each gene occurs and used a *t*-test to compare the values of the segments identified within subjects classified into the angiogenic subtype to the segments identified within subjects classified into the non-angiogenic subtype. To determine the overall differential-CNV for a set of target genes, we compared the resulting t-statistic value for the set of genes targeted by a particular transcription factor to all other genes [110]. Values for the differential-CNV of each gene are included in Additional file 4: Dataset S3.

### Characterizing the association of differential gene expression within classes of network genes

We wished to investigate how genes identified by our network model might respond to standard or other treatment protocols. Therefore, we analyzed publically-available experimental data measuring gene expression levels in response to various stimuli. For each experiment, we RMA-normalized raw CEL data deposited on the Gene Expression Omnibus using a custom-CDF to map to Entrez GeneIDs [63], selected samples that correspond to either a treatment or control experiment, and performed a *t*-test to quantify the expression differences between the treatment and control samples. Finally, we computed a summary statistic representing the significance of the association of this differential expression with genes in each of the “classes” defined by our subnetworks. Specifically, we calculate a “meta”-t-statistic and associated p-value by comparing the differential-expression t-statistic values for genes in a given network class to the t-statistic values for all other genes [110].

We also performed a randomization procedure to ensure that significant results identified in the above analysis is not accidental, and would not be observed for random “classes” of genes (Additional file 2: Figure S5). See the legend for Additional file 2: Figure S5 for more details.

### CMAP analysis

We downloaded the “raw” gene expression CEL files from the Connectivity Map website (http://www.broadinstitute.org/cmap/cel_file_chunks.jsp) and normalized these using fRMA [29]. This dataset contains the expression of approximately 12,000 genes before and after administration of 1,309 drugs in as many as 5 cell lines. We generated drug perturbation signatures by quantifying the differential gene expression, controlling for tissue type and batch effects using the following model:

$$ {G}_i={\beta}_{0,i}^d+{\beta}_{c,i}^d{C}^d+{\beta}_{t,i}^d{T}^d+{\beta}_{b,i}^d{B}^d,\forall i\in M $$

where variables are the same as those used for drug sensitivity signatures except for *C* ^*d*^, representing the concentration of drug *d* used to treat the cell lines, *T* ^*d*^, representing the tissue of the cell-line treated with drug *d*, and *B* ^*d*^, representing the batch of the array measuring the effect of drug *d*. The strength and significance of differential expression of gene *i* due to perturbation by drug *d* is given here by the term ${\beta}_{c,i}^d$ and its associated p-value (Student’s *t*-test). We defined the gene signatures for drug perturbations based on estimates for the coefficients of ${\beta}_{\mathrm{c},\mathrm{i}}^{\mathrm{d}}$ and their associated p-values.

### Code and materials for repeating the analysis in the paper

The PANDA implementation used to perform this analysis, data input files, output predicted networks, as well as a separate tool to perform edge-enrichment analysis on a pair of PANDA networks is available at http://sourceforge.net/projects/panda-net/.

## Additional files

Additional file 1: Dataset S1. (TranscriptionFactor_CombinatorialEnrichment.txt): The significance of co-targeting by all pairs of transcription factors in the various network classifications.
Additional file 2: Figure S1. Analysis demonstrating that network estimates are robust to the exact expression samples used. **Figure S2.** Analysis examining the effect of the prior motif structure on network estimates. **Figure S3.** Evaluation of how different protein-interaction databases affect PANDA’s predicted networks. **Figure S4.** Analysis examining the consequences of varying the threshold used to define the angiogenic and non-angiogenic subnetworks. **Figure S5.** Analysis examining whether the differential methylation and expression of target genes and gene classes might be observed by chance.
Additional file 3: Dataset S2. (TF_Statistics_Across_P-cutoffs.txt): A file containing the edge-enrichment and p-values for TF-differential-targeting between subnetworks identified across various P-cutoff values. The results included in this file were used to generate Additional file 2: Figure S4. The P-cutoff value of 0.8 was used to define the subnetworks used in the analysis shown in the main text (see Figure 2A).
Additional file 4: Dataset S3. (AllGenes_SubtypeInformation.txt): File containing various characteristics of the genes included in our network reconstruction. This includes (1) whether each gene was identified as a key TF or is a previously-identified biomarker for angiogenesis; (2) the network and expression classification of each gene (used for functional and CMAP analysis); and (3) the differential-expression, -methylation and –CNV of each gene between the subtypes.
