# Supplementary material for: A network model for angiogenesis in ovarian cancer
Source: BMC Bioinformatics. 2015 Apr 11;16:115. doi: 10.1186/s12859-015-0551-y (PMC4408593; doi:10.1186/s12859-015-0551-y)
Supplement: Additional file 2: Figure S1. — Analysis demonstrating that network estimates are robust to the exact expression samples used. Figure S2. Analysis examining the effect of the prior motif structure on network estimates. Figure S3. Evaluation of how different protein-interaction databases affect PANDA’s predicted networks. Figure S4. Analysis examining the consequences of varying the threshold used to define the angiogenic and non-angiogenic subnetworks. Figure S5. Analysis examining whether the differential methylation and expression of target genes and gene classes might be observed by chance. [file 12859_2015_551_MOESM2_ESM.pdf]

Supplemental Figure 1

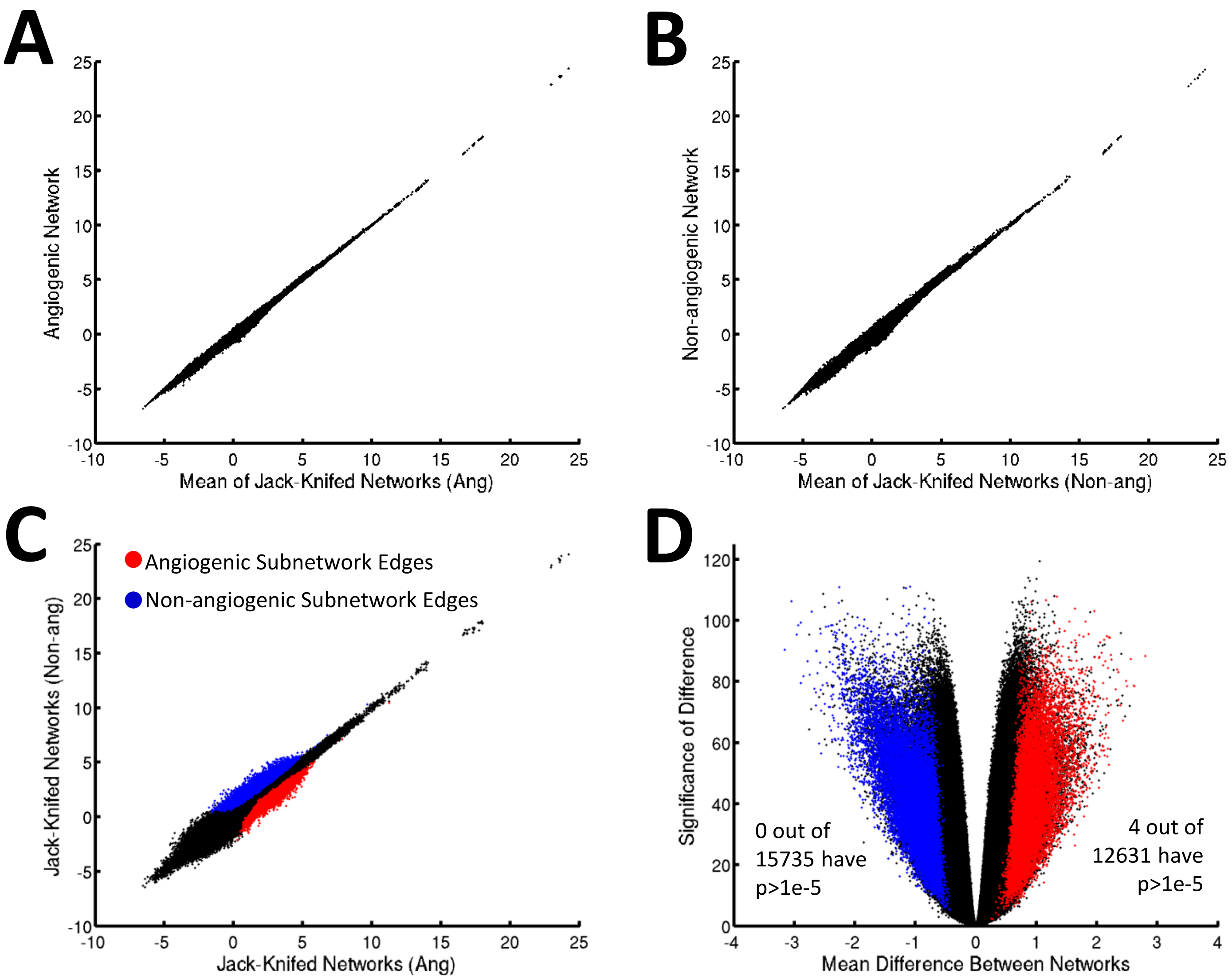

<u>Supplemental Figure 1:</u> Network estimates are robust to the exact expression samples used. In this analysis we selected 100 random angiogenic expression samples and 100 random non-angiogenic expression samples to build a "jack-knifed" angiogenic and non-angiogenic networks, respectively. We repeated this 100 times resulting in 100 "jack-knifed" angiogenic networks and 100 "jack-knifed" non-angiogenic networks. (A) The mean edge weight predicted across the jack-knifed angiogenic networks compared to the original weight predicted by PANDA for the angiogenic network built using all the samples. (B) The same thing for the non-angiogenic networks. (C) The mean edge weight across the jack-knifed angiogenic networks compared the mean edge weight across the jack-knifed non-angiogenic networks. The *original* identified angiogenic and non-angiogenic subnetworks are shown on the image in red and blue, respectively. Note that this image is nearly indistinguishable from Figure 1B in the main manuscript. (D) The difference in the mean edge weights across the angiogenic and non-angiogenic jack-knifed networks compared to the p-value of the statistical significance of this difference, as measured by an un-paired two sample t-test. The original edges identified as belonging to the angiogenic subnetwork and non-angiogenic subnetwork are shown in red and blue, respectively. Note that practically all of these edges are considered significantly different between the networks.

Supplemental Figure 2

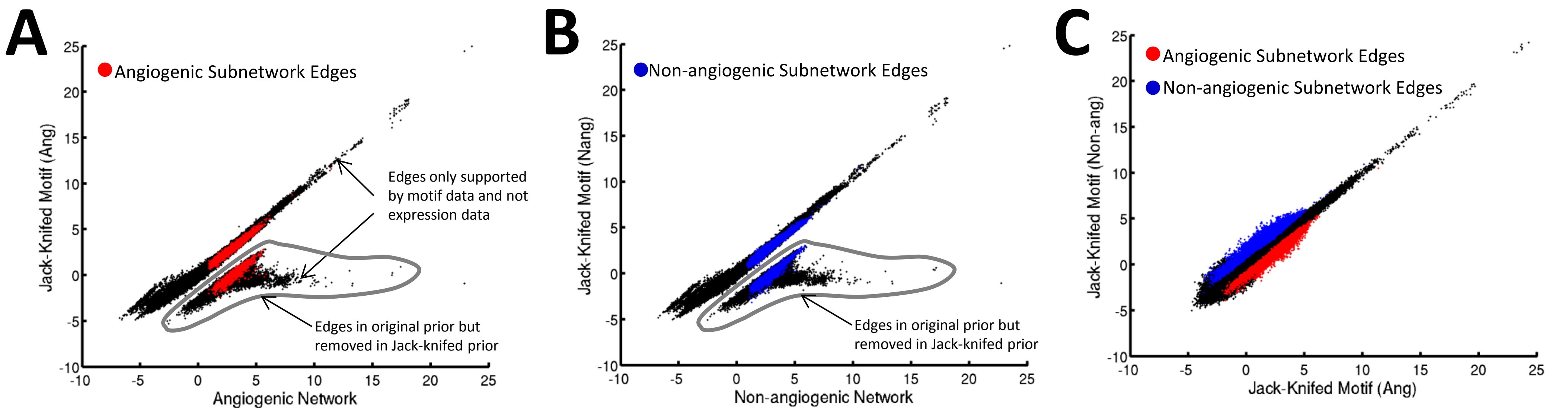

D

	1	2	3	4	5	6	7	8	9	10
NFKB1	5.27e-04	1.42e-03	7.60e-04	4.87e-03	8.62e-03	**1.86e-04**	**9.43e-06**	**2.45e-04**	1.63e-04	**2.51e-04**
ARID3A	**5.24e-53**	**1.79e-53**	**3.42e-51**	**1.17e-42**	**3.89e-33**	**1.03e-28**	**1.50e-40**	**2.72e-34**	**2.24e-59**	**2.35e-44**
SOX5	**5.19e-29**	**7.99e-36**	**1.01e-30**	**1.00e-30**	**2.10e-17**	**1.74e-23**	**1.57e-16**	**3.61e-22**	**2.17e-20**	**6.29e-28**
TFAP2A	**2.17e-11**	**1.99e-18**	**1.55e-08**	**8.95e-16**	**1.28e-11**	**5.54e-21**	**1.08e-08**	**5.05e-17**	**1.98e-12**	**1.14e-11**
NKX2-5	**1.95e-04**	**2.45e-07**	**3.49e-04**	**3.82e-04**	3.45e-02	1.13e-02	**6.90e-04**	1.12e-03	2.46e-02	3.34e-02
PRRX2	4.30e-08	**4.73e-23**	**1.66e-27**	**2.72e-21**	1.28e-15	**4.75e-22**	**5.38e-26**	3.83e-17	**4.62e-30**	**2.22e-26**
AHR	7.83e-18	**2.64e-32**	3.29e-20	2.74e-22	**2.14e-31**	1.34e-19	**2.72e-29**	1.12e-22	**6.00e-28**	**1.73e-24**
SPIB	**1.44e-06**	**1.45e-07**	**6.37e-09**	**9.97e-07**	**3.69e-09**	**2.41e-06**	**8.00e-06**	**8.06e-05**	**1.23e-09**	**4.02e-08**
MZF1	**1.12e-08**	**3.67e-10**	**7.00e-14**	**3.56e-17**	**6.49e-10**	**3.52e-12**	**4.82e-14**	**1.39e-11**	**1.46e-07**	**2.36e-14**
BRCA1	1.39e-03	1.75e-03	6.48e-03	4.72e-03	6.03e-03	5.12e-02	5.66e-02	3.73e-02	1.79e-02	9.26e-02
**Additional Identified TFs**	NFIC NKX3-1	ETS1 GATA3 NFIC	(none)	(none)	GATA3	(none)	GATA3	(none)	(none)	ETS1

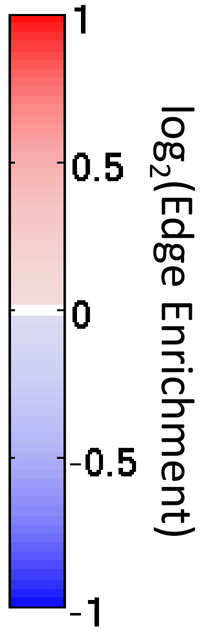

Supplemental Figure 2: Effect of the prior motif structure on network estimates. To evaluate the effect of the motif structure on the predicted network, we removed 10% of edges from our prior network and re-ran PANDA to integrate this jack-knifed motif prior with the protein interaction data and the angiogenic and non-angiogenic expression data, resulting in two "new" angiogenic and non-angiogenic networks. (A) and (B) show the originally-predicted angiogenic and non-angiogenic networks plotted against the networks predicted using the jack-knifed motif prior. There are two "clouds" of edges. Along the diagonal are the edges with the same assignment in the two network reconstructions (of either in the motif prior or not in the motif prior). These edges are highly similar in weight and we can conclude that removing other edges has only some minimal effect on the prediction of these remaining edges. A second cloud, shifted to the right and "under" the diagonal represents the 10% of edges that were in the original motif prior but excluded when reconstructing the network. This cloud appears to be made up of two groups of edges. One group runs parallel to the diagonal while the other runs parallel to the x-axis. The former group are edges whose prior prediction in modified by the expression data when run through PANDA, either increasing or decreasing in weight. The latter are edges for whom the expression data gives no additional information, and thus whose weight is determined by the local motif structure. The edges that compose the identified angiogenic and non-angiogenic subnetworks are colored red and blue and shown on figure (A) and (B) respectively. We note that the group of edges only influenced by the motif data were not included as a part of our subnetwork analysis. On the other hand, edges that were included in our subnetworks tend to be some of the highest-weight edges among those who had motif evidence in the original prior but for whom that evidence was removed in the jack-knifed motif. Finally, (C) shows the edge weight predicted in the angiogenic subtype versus the non-angiogenic subtype using the jack-knifed motif data. Again, we plot the *original* identified angiogenic and non-angiogenic subnetworks in red and blue, respectively, and find results highly consistent with that using the original motif prior. We repeated this analysis using ten different jack-knifed motif priors and observe similar results for (A)-(C) each time. (D) For the ten "key TFs" identified in the main text, we show edge enrichment and p-values calculated by comparing the angiogenic versus non-angiogenic network comparisons generated using each of the different jack-knifed motif priors. Transcription factors that meet the criteria for being identified as a "key TF" are noted by bolding their p-values and any additional transcription factors identified as a "key TF" in each comparison are listed. Overall there is very high similarity between the transcription factors identified from networks generated using the jack-knifed motif priors and those generated with the original "full" motif network prior.

Supplemental Figure 3

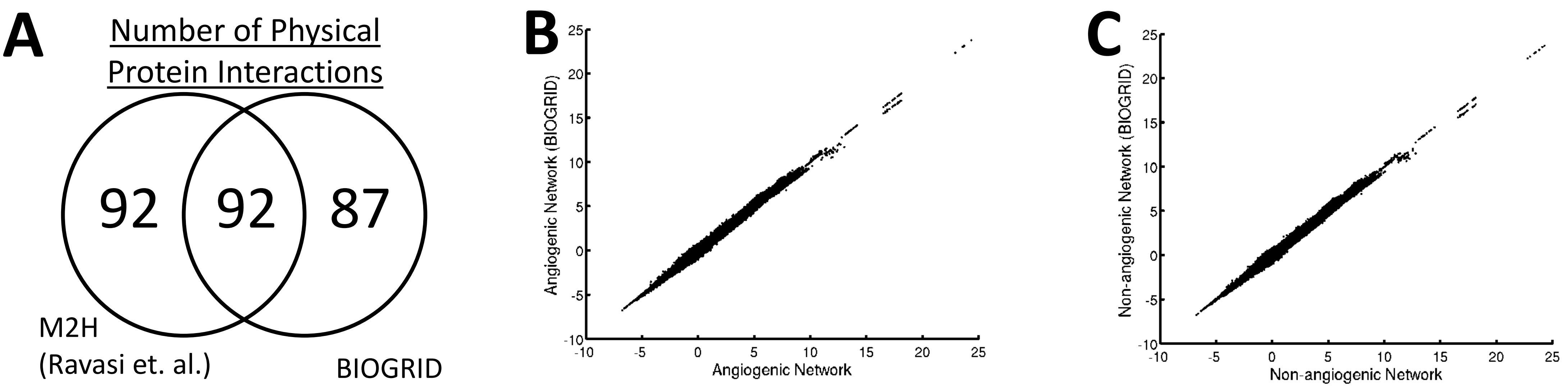

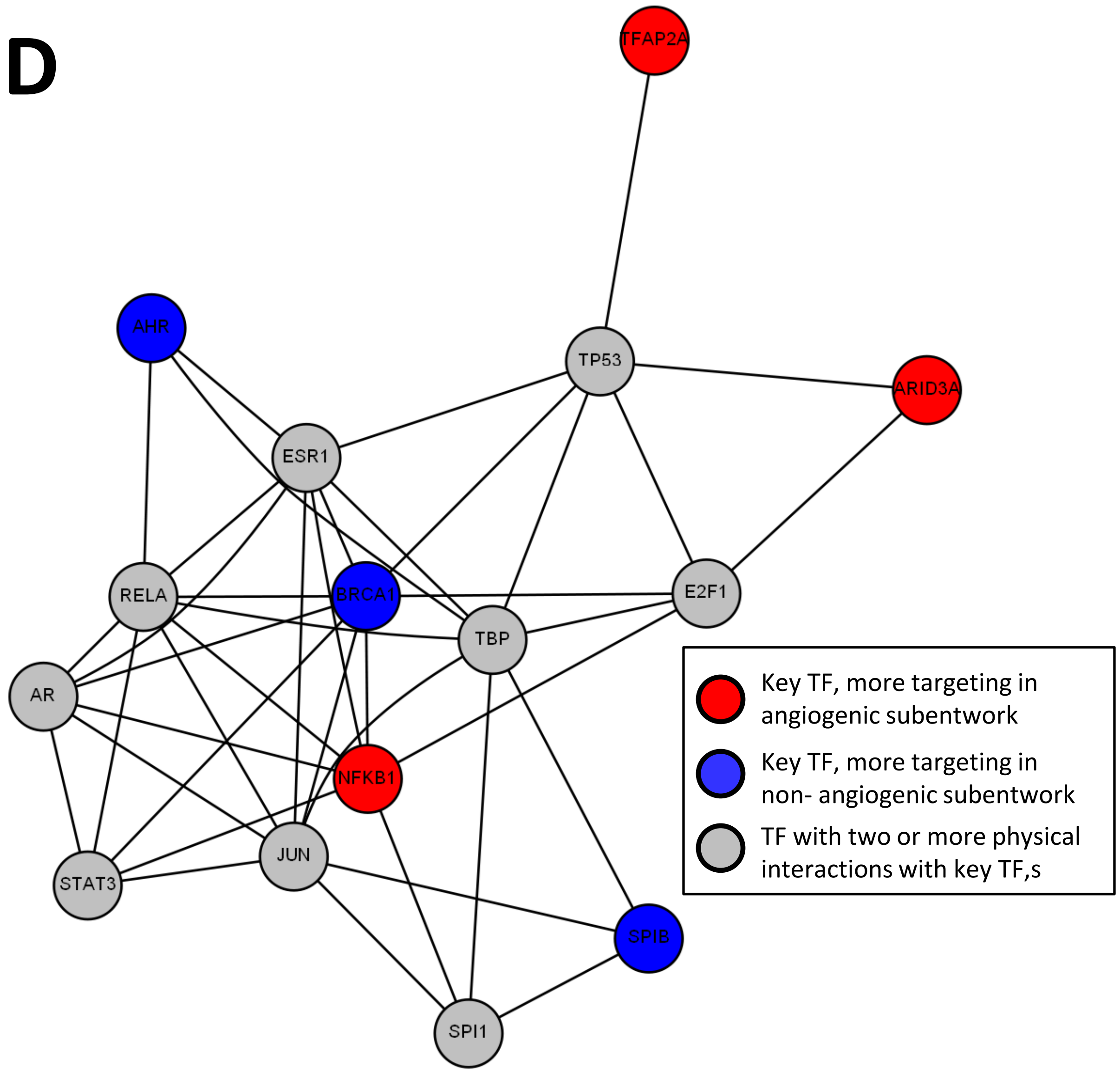

Supplemental Figure 3: Evaluation of how including protein-interactions effect PANDA's predicted networks. We evaluated two potential PPI databases in constructing PANDA networks, one published in Ravasi et. al. and used to build the networks in the main text, and the other from BIOGRID. To construct the BIOGRID PPI for this analysis we selected all physical or direct protein interactions between human transcription factors that were included in our motif prior. (A) The overlap in the number of unique interactions from each database, after matching against the human TFs included in the motif scan. (B-C) The edge weight predicted by PANDA when using the Ravasi et. al. PPI versus the one from BIOGRID for either the (B) angiogenic or (C) non-angiogenic network reconstruction. In both cases the weights predicted using the two different databases are highly similar, suggesting that PANDA's networks are not sensitive to the PPI database used. (D) To better appreciate the how the included PPIs may have contributed to the predicted network models, we show the local subnetwork around our ten "key" transcription factors (Figure 2A in the main text), by selecting any protein that had two or more physical interactions with one of the ten key TFs. We find several interesting proteins are included in this subnetwork, including ESR1, which has an interaction with both BRCA1 and AHR, AR, which interacts with both BRCA1 and NFKB1, and TP53, which interacts with TFAP2A, ARID3A and BRCA1.

Supplemental Figure 4

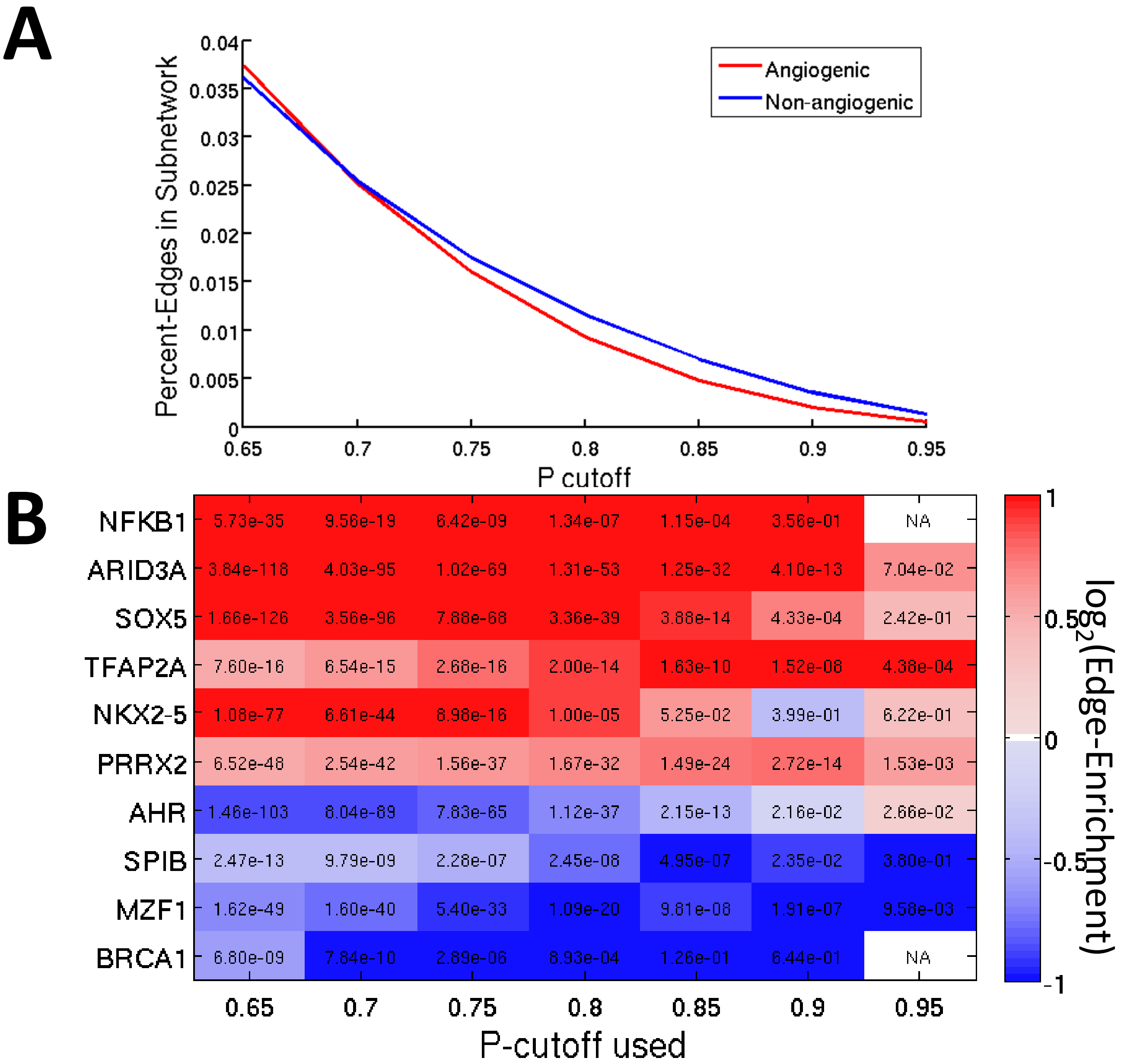

C

All TFs with k≥20, $p<10^{-3}$ and $E_{TF}>1.5$

0.65	0.7	0.75	0.8	0.85	0.9	0.95
AHR, ARID3A, ARNT, BRCA1, ELF5, ELK4, FOXC1, FOXD1, FOXO3, IRF1, MYB, MYC, MZF1, NFKB1, NFYA, NKX2-5, NKX3-1, NR4A2, PAX2, PDX1, PLAG1, SOX5, ZFX, ZNF143,	AHR, ARID3A, ARNT, BRCA1, ELK4, FOXD1, FOXO3, GATA3, MZF1, NFKB1, NFYA, NKX2-5, NKX3-1, NR4A2, PDX1, PLAG1, SOX5, TFAP2A,	AHR, ARID3A, BRCA1, ELK4, MZF1, NFKB1, NFYA, NKX2-5, NKX3-1, PRRX2, SOX5, TFAP2A,	AHR, ARID3A, MZF1, NFKB1, NKX2-5, PRRX2, SOX5, SPIB, TFAP2A,	ARID3A, EGR1, MZF1, NFIC, PRRX2, SOX5, SPIB, TFAP2A,	ARID3A, EGR1, ETS1, NFIC, PRRX2, SOX5, TFAP2A,	SP1

Supplemental Figure 4: The effect of the threshold used to define the angiogenic and non-angiogenic subnetworks. (A) A plot of the percentage of all edges that would be assigned to each subnetwork based on the threshold (P-cutoff) used. In the main text we used P=0.8 as it resulted in approximately 1% of all edges being assigned to each subnetwork. (B) The edge-enrichment and p-value significance of the ten "key" transcription factors described in the main text for each P-cutoff value. There is very high consistency across the cutoffs. For the very highest P-cutoff value (P=0.95)) the network becomes so sparse that some TFs have no outgoing edges in the identified subnetworks and the edge-enrichment and p-values calculations cannot be made . (C) The transcription factors that would be identified as "key" transcription factors at each cutoff, meaning they have more than twenty total interactions, edge-enrichment>1.5 (or less than 1/1.5) and $p<10^{-3}$. More transcription factors are called as the value of P decreases, largely because higher density in the subnetworks gives greater confidence (and thus lower p-values) in the differences.

Supplemental Figure 5

A Differential-expression of Random Gene Sets

NFKB1	ARID3A	SOX5	TFAP2A	NKX2-5	PRRX2	AHR	SPIB	MZF1	BRCA1
-0.50417 (-0.13198 +/- 1.0364)	9.7357 (-0.035378 +/- 1.0516)	7.1127 (-0.01064 +/- 1.026)	2.6717 (0.090721 +/- 1.0499)	5.7972 (-0.20419 +/- 1.0995)	2.3584 (0.18618 +/- 0.94001)	1.7488 (-0.06234 +/- 1.0786)	3.1867 (-0.035276 +/- 0.8989)	15.952 (0.11833 +/- 1.1941)	13.4758 (-0.15771 +/- 1.1369)

B Differential-methylation of Random Gene Sets

NFKB1	ARID3A	SOX5	TFAP2A	NKX2-5	PRRX2	AHR	SPIB	MZF1	BRCA1
0.39122 (-0.19648 +/- 0.95722)	-4.9483 (0.015734 +/- 0.93342)	-2.6371 (-0.10878 +/- 0.97629)	0.90975 (-0.034286 +/- 1.041)	-4.2065 (0.09715 +/- 1.012)	-4.9134 (0.074769 +/- 0.93371)	1.835 (-0.10746 +/- 1.0137)	2.8781 (-0.047713 +/- 1.0427)	1.9986 (0.075866 +/- 1.0006)	-0.56063 (-0.12404 +/- 1.0139)

C Drug Differential-Expression in Random Classes

	A+	A-	A+;N-	N+;A-	N-	N+
Drugfree Medium 16/0 hr (A2780)	0.51344 (0.14541 +/- 1.0451)	0.63606 (-0.014937 +/- 0.89329)	0.089376 (-0.023857 +/- 1.0131)	1.4612 (-0.018258 +/- 0.98178)	-1.3764 (-0.022301 +/- 1.0325)	0.80657 (-0.10546 +/- 0.97911)
cisplatin +/- (A2780)	-0.36681 (-0.013343 +/- 0.91445)	-2.6487 (-0.14423 +/- 0.95272)	2.5655 (0.11572 +/- 1.038)	-2.8026 (-0.19072 +/- 0.97846)	5.7629 (-0.0053696 +/- 1.0057)	-2.2566 (0.23714 +/- 0.90095)
oxaliplatin +/- (A2780)	0.48711 (0.024369 +/- 0.99471)	-3.3633 (-0.054112 +/- 0.91955)	3.9467 (0.079951 +/- 1.0605)	-4.2139 (-0.10499 +/- 0.84925)	6.797 (-0.018372 +/- 0.99152)	-0.41101 (0.072127 +/- 0.92669)
Sorafenib +/- (ER+ breast cancer tumors)	-10.1067 (0.060722 +/- 0.92584)	5.329 (0.22649 +/- 0.9979)	-6.6407 (0.083421 +/- 0.97616)	6.306 (-0.060386 +/- 0.93761)	-9.851 (0.20223 +/- 0.96464)	6.8804 (-0.099358 +/- 0.94956)
HIFa -/+ (HUVEC)	-3.898 (0.07241 +/- 1.0114)	0.023624 (0.10601 +/- 1.0669)	-0.73094 (0.12136 +/- 0.87093)	2.2517 (-0.17264 +/- 1.0542)	-1.8102 (0.21458 +/- 0.80333)	1.6523 (-0.13012 +/- 1.0711)
HIFa -/+ (multiple myeloma, JJN3)	-2.0483 (0.066126 +/- 0.97962)	1.1651 (-0.080214 +/- 1.0505)	-0.89149 (-0.0091086 +/- 1.0071)	-0.53407 (-0.10443 +/- 1.0162)	0.79274 (0.027045 +/- 1.0665)	0.9192 (0.077333 +/- 1.0353)
TCDD +/- (Hepatocytes)	-4.1654 (-0.015356 +/- 1.0081)	4.2587 (0.2041 +/- 0.96667)	-5.127 (-0.083732 +/- 1.1107)	0.804 (-0.21625 +/- 1.1577)	-4.4007 (-0.027406 +/- 1.0041)	-0.33919 (0.067258 +/- 1.0246)
TCDD +/- (CD34+ hemotopoetic cells)	-4.1838 (0.25572 +/- 0.93497)	2.523 (-0.088989 +/- 1.0326)	1.1524 (0.12895 +/- 0.94239)	0.53245 (-0.024427 +/- 0.94811)	-0.22284 (-0.10176 +/- 0.99008)	0.65574 (-0.096151 +/- 1.0454)
5-aza +/- (OC)	-4.4857 (0.078364 +/- 0.9815)	1.7762 (-0.027783 +/- 1.0085)	-1.5857 (-0.085863 +/- 0.9408)	2.2683 (-0.19849 +/- 1.0853)	-1.9251 (-0.2715 +/- 1.0784)	-1.8372 (0.20453 +/- 1.0347)

Supplemental Figure 5: Evaluate of Differential Expression and Methylation in Random Gene Sets and Classes. To evaluate whether we would have observed the same differential-expression and differential-methylation patterns by chance we performed several different analyses. First, for each transcription factor, we created 100 random sets of genes, each of equal size to the number of target genes identified by our network analysis. In (A) we report the t-statistic of differential-expression for the original target gene set for each TF, as well as the average and standard deviation for the 100 random sets of genes. In (B) we repeat the analysis, but evaluating differential-methylation. Next, we create 100 random classes of genes, again of the same size as the original six classes we defined by combining our network findings with differential-gene expression. In (C) we report the t-statistic of the differential-expression of genes in each of the selected datasets for the network and expression defined classes of genes, as well as the average and standard deviation for each of the 100 random classes.
